# Characteristics of Peripheral Lymphocyte Subsets in Patients With Acute-On-Chronic Liver Failure Associated With Hepatitis B

**DOI:** 10.3389/fmed.2021.689865

**Published:** 2021-07-27

**Authors:** Juan Li, Chun-Hua Hu, Yi Chen, Mi-Mi Zhou, Zhi-Jie Gao, Meng-Jun Fu, Jing Wang, Jian-Zhou Li, Tian-Yan Chen, Ying-Ren Zhao, Ying-Li He

**Affiliations:** ^1^Department of Infectious Diseases, School of Medicine, First Affiliated Teaching Hospital, Xi'an Jiaotong University, Xi'an, China; ^2^School of Medicine, Institution of Hepatology, First Affiliated Teaching Hospital, Xi'an Jiaotong University, Xi'an, China; ^3^Shaanxi Clinical Research Center of Infectious Diseases, Xi'an, China

**Keywords:** lymphocyte subsets, hepatitis B virus, acute-on-chronic liver failure, immune response, flow cytometry

## Abstract

**Background and Aims:** Acute-on-chronic liver failure (ACLF) is a rare, but dramatic clinical syndrome. There is substantial evidence suggesting that immunity-mediated inflammation plays an important role in HBV-ACLF. Our aim was to characterize the proportion and cell counts of peripheral blood lymphocyte subsets in acute-on-chronic liver failure patients caused by HBV infection.

**Methods:** One hundred and seventeen patients were enrolled in this study, including those with HBV-related ACLF (HBV-ACLF; *n* = 70), and HBV related non-ACLF patients (HBV non-ACLF; *n* = 47). Demographics, clinical and laboratory data at hospital admission were retrospectively analyzed. The percentage and cell count of peripheral lymphocyte subsets were evaluated by flow cytometry. Comparison analysis was performed by *t*-test or non-parametric Mann–Whitney *U*-test. Actuarial probabilities of death were calculated by the Kaplan-Meier method.

**Results:** Both circulating lymphocyte count and lymphocyte percentage were significantly reduced in patients with HBV-ACLF (*P* < 0.001). The CD8^+^ T cell, CD4^+^ T cell, and CD16^+^CD56^+^ NK cell counts were significantly decreased in HBV-ACLF. Consistently, flow cytometric analysis showed that CD8^+^ T cell counts were significantly decreased in non-survivors, while no significant differences were found in CD4^+^ T cell, CD19^+^ B cell, or CD56^+^CD16^+^ NK cell counts. Furthermore, the group with the lower CD8^+^ T cell count displayed a significantly higher mortality rate compared with the group with the higher CD8^+^ T cell count.

**Conclusions:** The abnormal prevalence of lymphocyte subsets may be important in the pathogenesis of HBV-ACLF. The decrease in CD8^+^ T cell counts may be related to poor survival in HBV-ACLF patients.

## Introduction

Acute-on-chronic liver failure (ACLF) is a rare, but dramatic clinical syndrome characterized by massive hepatocyte death leading to multiorgan failure in patients with pathological damage caused by chronic liver disease (1). The most common cause of ACLF in China is chronic hepatitis B virus (HBV) infection (2). ACLF results in an extremely high mortality rate due to its unclear pathogenesis and the lack of effective treatment approaches (3). There is substantial evidence to suggest that immunity-mediated inflammation plays an important role in HBV-ACLF. The neutrophil–lymphocyte ratio (NLR) and lymphocyte–monocyte ratio (LMR), which reflect systemic inflammation, are valuable prognostic markers in ACLF patients (4–8).

Several reports on the immune pathogenesis of chronic HBV infection have suggested that CD8^+^ T cells, CD4^+^ T cells, and NK cells as well as cytokines participate in the development of liver injury (9–13). A recent study (13) revealed that interleukin 21 (IL-21) enhanced the antiviral responses of CD8^+^ T cells in chronic HBV infection. Zou et al. (14) characterized lymphocyte subsets in peripheral blood and liver tissue, and reported that the abnormal distribution of circulating and liver-infiltrating immune competent cells may be an important factor for the development of HBV-related ACLF. Dong et al. (15) demonstrated that HBV-ACLF patients displayed immune disorders from the perspective of adaptive immunity, which were characterized by a reduction in the number of CD4^+^ T lymphocytes. Similarly, another study on peripheral lymphocytes reported the exhaustion of differentiated CD4^+^ T cells from the circulation in HBV-ACLF patients compared to patients without ACLF (7). Our previous study found that Natural Killer Group 2A (NKG2A) expressed on peripheral CD3^−^CD56^+^ NK cells and CD3^+^CD8^+^ T cells played a key negative regulatory role in the progress of HBV-related ACLF (16). However, there are still insufficient data to show the overall characteristics of immune cell status in HBV-related ACLF patients and their association with prognosis. Therefore, this study focused on the proportion and number of peripheral blood lymphocyte subsets in HBV-related ACLF patients to characterize changes in immune status and clarify the correlation between liver injury and its immunological characteristics.

## Materials and Methods

### Study Population

The data from patients with chronic HBV infection who were admitted from July 2013 to November 2019 in the First Affiliated Hospital of Xi'an Jiaotong University, the largest general hospital in northwest China under the direct administration of the Chinese Ministry of Health, were retrospectively collected, including 70 patients with HBV-ACLF and 47 patients with HBV-non-ACLF. HBV-non-ACLF referred to patients with chronic hepatitis B, or HBV-related compensated cirrhosis, who had abnormal liver function due to chronic HBV infection, but do not meet the diagnostic criteria of ACLF. ACLF was diagnosed according to the recommendation by the Asian Pacific Association for the Study of the Liver (APASL) (17): serum bilirubin ≥5 mg/dL, an international normalized ratio (INR) ≥1.5 or prothrombin activity <40%, recent development of complications such as hepatic encephalopathy, or an abrupt and obvious increase in ascites, spontaneous bacterial peritonitis, or hepatorenal syndrome. Briefly, CHB patients were HBsAg positive for more than 6 months and may have exhibited signs or symptoms of hepatitis and abnormal liver function. Cirrhosis was diagnosed based on liver biopsy (if available), or the combination of clinical symptoms, laboratory tests, and CT/MRI scan. HBV-non-ACLF refers to patients who had alanine aminotransferase or aspartate aminotransferase levels >2 times the upper limit of normal (ULN) (40 U/L) and a total bilirubin level <5 ULN (1 mg/dL).

Patients were excluded if they had any of the following conditions: (1) malignancies, such as hepatocellular carcinoma; (2) concurrent hepatitis A, hepatitis C, hepatitis D, hepatitis E, Epstein–Barr virus, or other virus infections; (3) with one or more additional known primary or secondary causes of liver disease, other than hepatitis B. All patients received standard care and treatments as recommended by the guidelines.

This retrospective study was conducted in accordance with the Declaration of Helsinki and the protocol was approved by the Ethics Committee of the First Affiliated Hospital of Xi'an Jiaotong University.

### Laboratory Examinations

Patients and controls underwent routine laboratory evaluations for liver diseases, including clinical assessments, complete blood count, liver function tests (alanine aminotransferase, total bilirubin, serum albumin), renal function (serum creatinine), electrolytes (serum sodium, potassium) and coagulation (international normalized ratio), HBV DNA load (COBAS TaqMan, lower detection limit 20 IU/mL). The tests were performed at the central laboratory in the hospital. Model for end-stage liver disease (MELD) score and MELD-Na score were used to assess disease severity and calculated as previously described (18).

### Flow Cytometry for Detection of Lymphocytes Subsets

One hundred microliters of blood sample were stained with 10 μL of fluoroisothiocyanate (FITC)-conjugated CD4, PE-conjugated CD8, Per-CP-conjugated CD3, APC-conjugated CD19, and Multitest CD16^+^CD56 reagent (Beckman Coulter). Then incubation for 15 min in the dark and red blood cells lysis were done. After washing, the cells were resuspended in Cytomics FC 500 (Beckman Coulter) flow cytometric analysis was done with Cell Quest software. Lymphocytes were defined with their forward and side scatter characteristic. T lymphocytes were identified (CD3^+^), and then subdivided into CD4^+^ or CD8^+^ populations. B lymphocyte (CD3^−^CD19^+^) and natural killer cell (CD3^−^CD56^+^CD16^+^) numbers and percentages were also determined.

### Statistical Analysis

Statistical analysis was performed using SPSS 23.0 for Windows (SPSS, Chicago, IL, USA), with graphs drawn using GraphPad Prism 8.0 (GraphPad, La Jolla, CA, USA). Quantitative data were expressed as mean ± standard deviation (SD) or median (interquartile range, IQR), and the categorical data were expressed as the number (percentage). *T*-test or the non-parametric Mann–Whitney *U*-test was used where appropriate. A Pearson's Chi-square or Fisher's exact test was performed to compare qualitative data. Actuarial probabilities of death during follow-up were calculated by the Kaplan-Meier method and compared by log-rank test. Results with a two-tailed *P*-value of <0.05 were considered statistically significant.

## Results

### Demographic and Clinical Characteristics of the Study Population

The clinical and biochemical characteristics of the enrolled groups were summarized in Table 1. There were no significant differences in age, gender, creatinine, HBeAg positive ratio and HBV DNA load between the two groups. As expected, HBV-related ACLF patients displayed higher levels of ALT, AST, total bilirubin and INR, but lower levels of albumin and decreased platelet count compared with non-ACLF group. The 28-day mortality rate in the ACLF group was 30.0% (21/70), which was significantly higher than that in the non-ACLF group.

**Table 1:** Demographic and clinical parameters of the study population.

**Parameters**	**HBV ACLF**	**HBV non-ACLF**	***P-*value**
	**(*n* = 70)**	**(*n* = 47)**	
Age (years)	38 (28–49)	39 (29–51)	0.885
Gender (m/f)	57/13	34/13	0.246
ALT (U/L)	193.00 (88.66–689.80)	123.15 (41.28–670.73)	0.018
AST (U/L)	189.00 (90.08–782.50)	58.75 (44.70–413.83)	<0.001
TBIL (μmol/L)	229.60 (154.47–372.10)	16.80 (10.20–37.96)	<0.001
Albumin (g/L)	32.86 ± 1.05	36.70 ± 1.62	<0.001
Creatinine (μmol/L)	56.40 (49.70–64.60)	60.35 (45.55–67.75)	0.748
PT (s)	22.10 (19.45–27.45)	13.65 (13.18–14.90)	<0.001
INR	1.83 (1.54–2.34)	1.12 (1.04–1.23)	<0.001
HBeAg positive	34 (48.57)	18 (38.30)	0.273
lgHBsAg (lg IU/mL)	2.68 ± 0.17	3.13 ± 0.25	0.108
lgHBV DNA (lg IU/mL)	5.10 (3.87–6.03)	4.21 (3.06–6.18)	0.237
WBC (×10^9^/L)	5.37 (4.48–7.29)	5.15 (3.83–5.85)	0.027
PLT (×10^9^/L)	92.98 ± 8.05	145.94 ± 17.66	<0.001
Lymphocyte count (×10^9^/L)	1.23 ± 0.08	1.70 ± 0.16	<0.001
Lymphocytes percentage (%)	22.56 ± 1.87	33.50 ± 2.21	<0.001
Mortality, *n* (%)	21 (30.0)	0 (0)	<0.001

*Data are expressed as mean ± standard deviation (SD), number (percentage), or median (interquartile range). ACLF, acute-on-chronic liver failure; ALT, alanine aminotransferase; AST, aspartate Aminotransferase; TBIL, total bilirubin; INR, international normalized ratio; HBV, hepatitis B virus, HBsAg, HBV surface antigen WBC, white blood cell count; PLT, platelet count*.

### Peripheral Lymphocyte Subsets in Patients With HBV-Related ACLF and Non-ACLF

**Figure 1:**
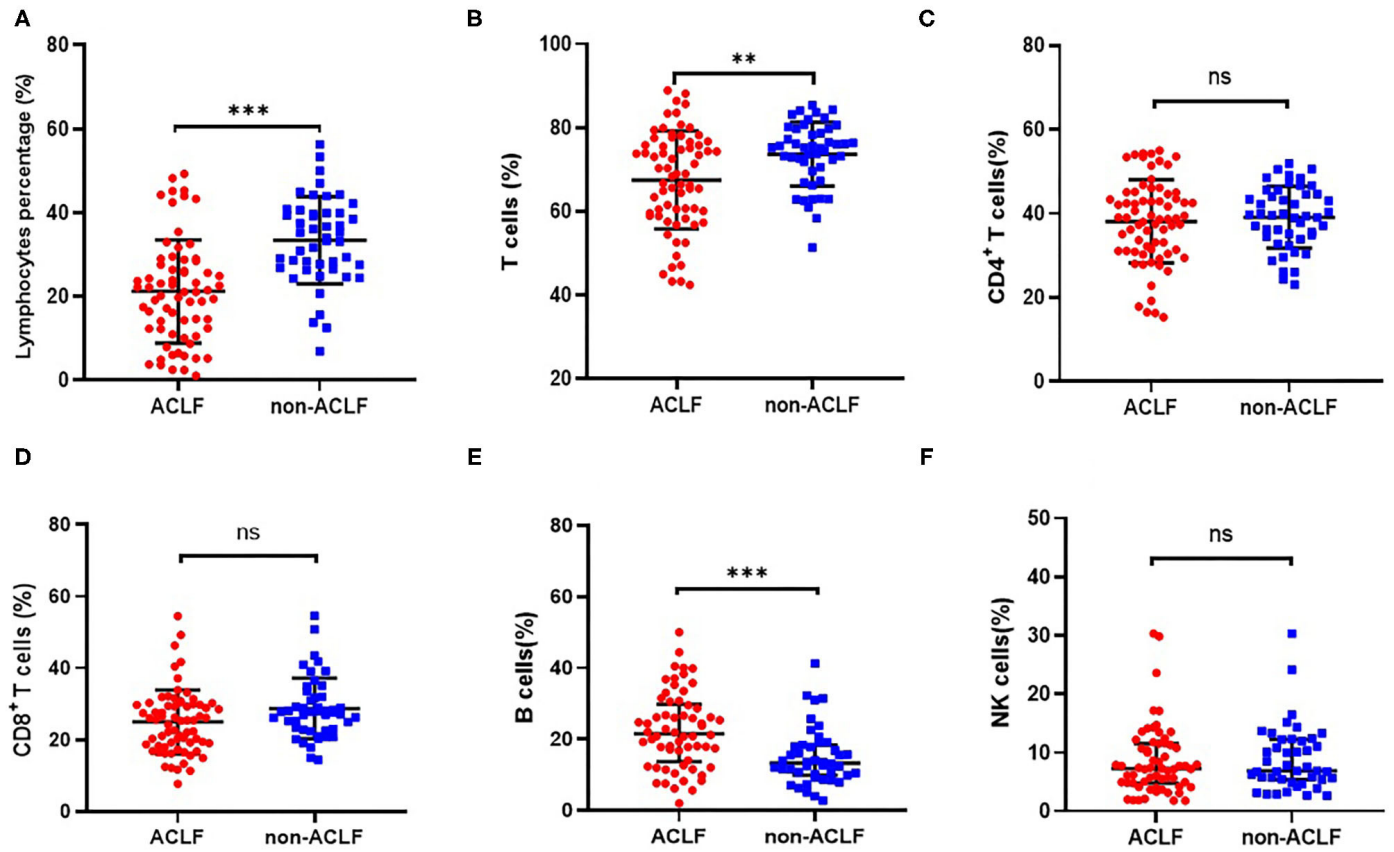
Comparison of the proportion of lymphocyte **(A)**, CD3^+^ T cells **(B)**, CD4^+^ T cells **(C)**, CD8^+^ T cells **(D)**, CD19^+^ B cells **(E)** and CD16^+^CD56^+^ NK cells **(F)** in patients with hepatitis B-related acute-on-chronic liver failure (ACLF) (*n* = 70) and non-ACLF (*n* = 47), where the lines indicated the mean or median. **P < 0.01, ***P < 0.001.

**Figure 2:**
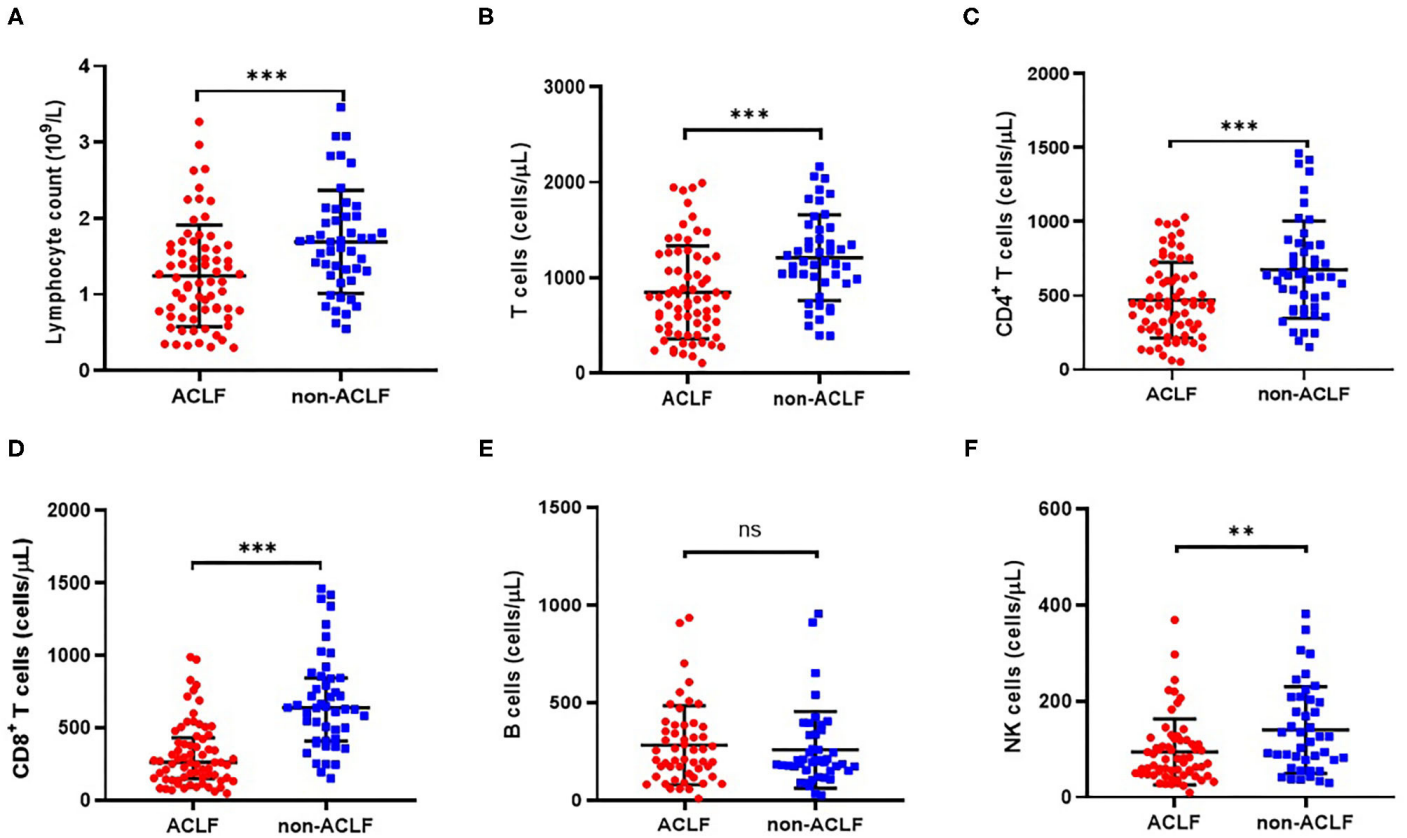
Comparison of cell counts of lymphocyte **(A)**, CD3^+^ T cells **(B)**, CD4^+^ T cells **(C)**, CD8^+^ T cells **(D)**, CD19^+^ B cells **(E)** and CD16^+^CD56^+^ NK cells **(F)** in patients with hepatitis B-related acute-on-chronic liver failure (ACLF) (*n* = 70) and non-ACLF (*n* = 47), where the lines indicated the mean or median. **P < 0.01, ***P < 0.001.

Compared with patients in the HBV non-ACLF group, both circulating lymphocyte count and lymphocyte percentage were significantly reduced in patients with liver failure (*P* < 0.001, Figures 1A, 2A). With regard to lymphocyte subset proportion, no significant differences were observed in CD4^+^ T cells, CD8^+^ T cells and CD16^+^CD56^+^ NK cells (Figures 1C,D,F). The proportion of CD3^+^ T cells in HBV-ACLF patients was significantly lower than that in the non-ACLF group (*P* = 0.003, Figure 1B), while the percentage of CD19^+^ B cells was significantly higher compared with the non-ACLF group (*P* < 0.001, Figure 1E).

Additionally, lymphocyte subset counts between the two groups were analyzed. Compared with the HBV non-ACLF group, the decrease in total lymphocyte count in ACLF patients (Figure 2A) was possibly due to a relative decrease in CD8^+^ T cell (Figure 2D), CD4^+^ T cell (Figure 2C), and CD16^+^CD56^+^ NK cell counts (Figure 2F). However, the lower number of CD19^+^ B cells in ACLF patients was not significant (Figure 2E).

### Comparison of Peripheral Lymphocyte Subsets Between Non-Surviving and Surviving ACLF Patients

To investigate whether peripheral lymphocyte subsets are correlated with short-term prognosis in HBV-related ACLF, a comparative analysis was performed between the surviving and non-surviving patients during a 28-day follow-up period. Of 70 ACLF patients, 21 patients died (mortality rate: 30.0%). Non-surviving patients showed higher total bilirubin, creatinine and INR at hospital admission (*P* = 0.007, *P* = 0.017 and *P* < 0.001, respectively, Table 2). In addition, the presence of encephalopathy (42.85 vs. 18.36%) and bacterial infection (85.71 vs. 57.14%) were both significantly higher in non-survivors compared with survivors (*P* = 0.032, *P* = 0.042, respectively, Table 2). Non-survivors showed a significantly higher MELD and MELD-Na score and lower lymphocyte percentage compared with survivors (*P* < 0.001, Table 2).

**Table 2:** Demographics, clinical data, and laboratory parameters in survivors and non-survivors of patients with HBV-related ACLF.

**Parameters**	**Survivors (*n* = 49)**	**Non-survivors (*n* = 21)**	***P-*value**
Age (years)	35.60 ± 1.83	52.78 ± 6.11	0.009
Gender (m/f)	42/7	15/6	0.159
ALT (U/L)	297.00 (105.83–746.33)	107.00 (82.50–450.90)	0.878
AST (U/L)	201.50 (88.25–809.00)	189.00 (101.75–755.05)	0.590
Cholesterol (mmol/L)	2.31 ± 0.10	1.76 ± 0.26	<0.001
TBIL (μmol/L)	244.88 ± 26.15	379.75 ± 62.51	0.007
Albumin (g/L)	31.95 (29.20–38.93)	29.24 (27.85–33.05)	0.039
Creatinine (μmol/L)	55.25 (48.05–60.15)	69.30 (55.20–83.36)	0.017
Sodium (mmol/L)	137.55 (135.00–139.00)	135.00 (129.50–137.75)	0.119
PT (s)	20.60 (17.85–23.85)	27.60 (24.90–39.25)	<0.001
INR	1.74 (1.46–1.95)	2.36 (2.06–3.50)	<0.001
HBeAg positive	23 (46.94)	11 (52.38)	0.676
lgHBsAg (lg IU/mL)	2.50 ± 0.20	3.20 ± 0.32	0.090
lgHBV DNA (lg IU/mL)	4.65 ± 0.31	6.13 ± 0.65	0.014
WBC (×10^9^/L)	5.22 (4.12–7.13)	7.37 (5.47–10.98)	0.006
PLT (×10^9^/L)	99.90 ± 10.02	82.11 ± 11.90	0.018
Lymphocyte count (×10^9^/L)	1.35 ± 0.10	1.01 ± 1.13	0.050
Lymphocytes percentage (%)	23.70 (16.95–32.72)	8.72 (5.05–21.85)	<0.001
Ascites, *n* (%)	20 (40.82)	13 (61.90)	0.105
Encephalopathy, *n* (%)	9 (18.36)	9 (42.85)	0.032
Bacterial infection, *n* (%)	28 (57.14)	18 (85.71)	0.042
PE/DPMAS, *n* (%)	18 (36.73)	9 (42.85)	0.630
Use of antibiotic, *n* (%)	28 (57.14)	18 (85.71)	0.042
MELD score	23.40 ± 0.99	29.44 ± 2.65	<0.001
MELD-Na score	22.90 ± 1.04	25.67 ± 2.04	0.001

*Data are expressed as mean ± standard deviation (SD), number (percentage) or median (interquartile range). ALT, alanine aminotransferase; AST, aspartate Aminotransferase; TBIL, total bilirubin; INR, international normalized ratio; WBC, white blood cell count; PLT, platelet count; PE, plasma exchange; DPMAS, double plasma molecular absorption system; MELD, model for end-stage liver disease*.

**Figure 3:**
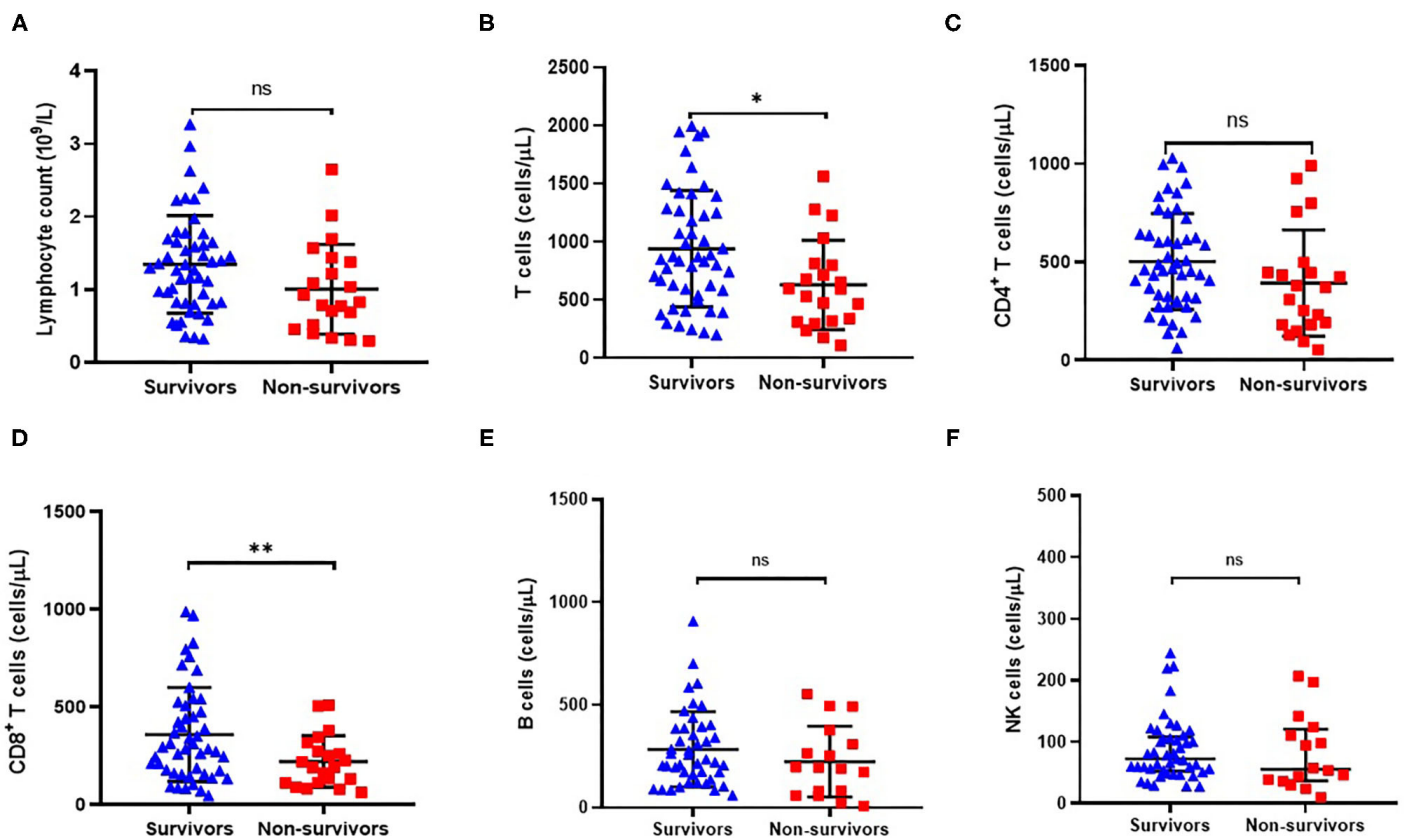
Comparison of peripheral absolute counts of lymphocyte **(A)**, CD3^+^ T cells **(B)**, CD4^+^ T cells **(C)**, CD8^+^ T cells **(D)**, CD19^+^ B cells **(E)** and CD16^+^CD56^+^ NK cells **(F)** in surviving (*n* = 49) and non-surviving (*n* = 21) patients with HBV-related ACLF. *P < 0.05, **P < 0.01.

As shown in Figure 3A, total lymphocyte count was lower in non-survivors compared with survivors (1.01 ± 1.13 vs. 1.35 ± 0.10) although the difference was not statistically significant (*P* = 0.050, Table 2). Since the absolute number of lymphocytes was already small in ACLF, it is of little sense to explore the proportion of lymphocyte subgroups between survivors and non-survivors. Consequently, we analyzed the absolute counts of lymphocyte subsets. Flow cytometric analysis showed that the CD8^+^ T cell count was significantly decreased in non-survivors (*P* = 0.003, Figure 3D), while no significant differences were found in CD4^+^ T cell, CD19^+^ B cell, or CD56^+^CD16^+^ NK cell counts (Figures 3C,E,F).

### Decreased CD8^+^ T Cell Count at Admission Was Correlated With Poor Prognosis in Patients With HBV-ACLF

**Figure 4:**
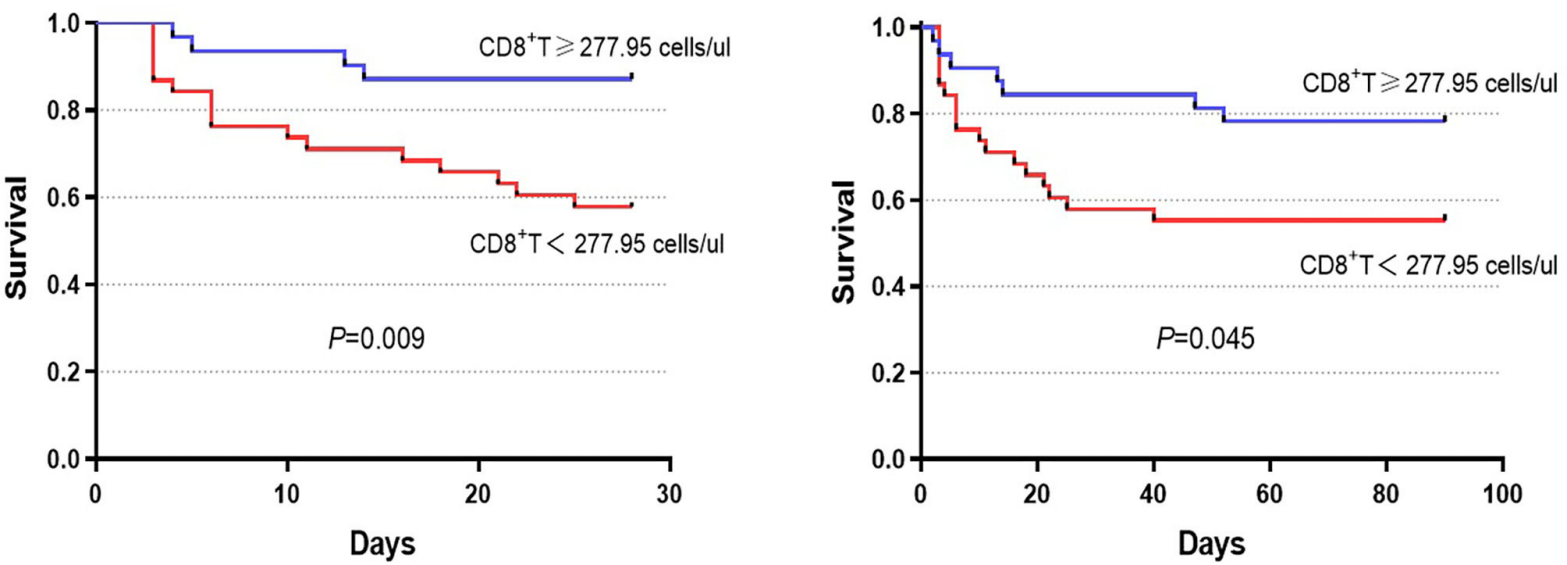
Survival was evaluated using Kaplan–Meier curves, and the statistics were compared by log-rank tests. Significant differences were found between the higher group (CD8^+^ T cell count ≥ 277.95 cells/μl) and the lower group (CD8^+^ T cell count <277.95 cells/μl) in 28-day (chi-square = 6.803, *P* = 0.009) and 90-day (chi-square = 4.015, *P* = 0.045) survival.

We further divided the patients into the lowest and highest groups according to the median of absolute numbers of peripheral blood lymphocytes to compare their short-term prognosis. The group with the lower CD8^+^ T cell count displayed a significantly higher mortality rate compared to the group with the higher CD8^+^ T cell count (42.9 vs. 17.1%, *P* = 0.019, Table 3). Similar results were found for CD3^+^ T cell counts, which may have been the relative result of reduced CD8^+^ T cell counts. With the median of the CD8^+^ T cell counts as the cut-off value (277.95 cells/μl), survival probability in patients with HBV-ACLF was shown in Figure 4. These results indicated that reduced CD8^+^ T cell counts might be related to poor prognosis in HBV-ACLF patients.

**Table 3:** Comparison of mortality rate between the higher and lower group divided by median values of lymphocyte count in HBV-related ACLF patients.

**Mortality (*n*, %)**	**Lower group**	**Higher group**	***P*-value**
CD3^+^ T cells (cells/μl)	15 (42.9)	6 (17.1)	0.019
CD4^+^ T cells (cells/μl)	14 (40.0)	7 (20.0)	0.068
CD8^+^ T cells (cells/μl)	15 (42.9)	6 (17.1)	0.019
CD19^+^ B cells (cells/μl)	13 (37.1)	8 (22.9)	0.192
CD56^+^CD16^+^ NK cells (cells/μl)	12 (34.3)	9 (25.7)	0.434

## Discussion

The results of the current study showed that the CD8^+^ T cell, CD4^+^ T cell, and CD16^+^CD56^+^ NK cell counts were significantly decreased in HBV-ACLF. Consistently, decreased CD8^+^ T cell counts was observed in non-survivors in comparison with survivors. Furthermore, the group with the lower CD8^+^ T cell count displayed a significantly higher mortality rate compared with the group with the higher CD8^+^ T cell count. The present results indicated that decreased CD8^+^ T cell counts might relate to the poor outcome in patients of HBV-ACLF.

It is known that chronic HBV infection lead to T cell exhaustion or dysfunction, resulting in the immunotolerance and viral persistence (19). The pathogenesis of HBV-ACLF is also a dysfunctional immune response caused by increased systemic inflammation and immune cell paralysis, which has been manifested in pre-existing liver diseases. Moreover, it was reported that immune disorders in ACLF were similar to sepsis, with abnormalities in the immune response, ranging from excessive inflammation to immune depression (20, 21). This study investigated the distribution characteristics of dominant peripheral lymphocyte subsets in patients with HBV-related ACLF. Consistent with previous findings (6, 8, 14), significant reductions were observed both in peripheral lymphocyte percentage and absolute number in ACLF patients compared with non-ACLF patients. We noted that the decrease in total lymphocyte counts in ACLF patients was possibly the result of a relative decrease in CD8^+^ T cell as well as CD4^+^ T cell and CD16^+^CD56^+^ NK cell counts (Figure 2, respectively). However, no significant difference was observed in the proportion of CD4^+^ T cells, CD8^+^ T cells and CD16^+^CD56^+^ NK cells between HBV-ACLF and non-ACLF patients. This observation could be explained by exhausted lymphocytes during liver failure. It seems that under the immunosuppressive condition of ACLF, the change in lymphocyte subset counts was more obvious than the proportion of these subsets. Notably, we found that the frequency of circulating CD19^+^ B cells in ACLF patients was significantly increased. A recent study demonstrated that HBV-ACLF patients had higher serum IgG, IgA, and IgM levels compared to CHB patients (22). Moreover, an overwhelming B cell response apparently centered in liver tissue was observed in patients with HBV-associated acute liver failure (23). These studies indicated that the B-cell immune response might play a role in the pathogenesis of HBV-related ACLF.

Importantly, we found that CD8^+^ T cell counts were significantly decreased in non-survivors compared with survivors (Figure 3D). Furthermore, the group with the lowest CD8^+^ T cell count displayed a significantly higher mortality rate compared to the group with the highest CD8^+^ T cell count, indicating that a reduction in CD8^+^ T cell count might be related to the poor prognosis of HBV-ACLF patients (Figure 4). Thus, circulating lymphocyte numbers could be a potential parameter for monitoring disease progression, especially CD8^+^ T cells. It is reasonable to believe that lower circulating lymphocytes in peripheral blood, like CD8^+^ T cells, increase the risk of infection and result in endotoxemia, which in turn, exacerbates inflammatory damage of liver tissue (13, 24–26). A recent study of ACLF patients reported that peripheral mononuclear myeloid-derived suppressor cell expansion suppressed T-cell proliferation and increased sensitivity to bacterial infections (27). In addition, previous studies reported that CD8^+^ T cells were more prone to undergoing apoptosis or clonal deletion, especially in the absence of activated CD4^+^ T cells (28, 29). Therefore, monitoring these circulating lymphocytes, especially CD8^+^ T cells, might help to predict the extent of liver damage.

There are however some limitations in our study. First, this is a retrospective review of a single-center experience. The current findings need to be confirmed in large, multicenter prospective studies. Second, we only investigated the prevalence of dominant peripheral lymphocyte subsets, and the cellular immune responses in liver tissue are unclear. Studying the histology of the liver may be interesting as changes in T cells in peripheral blood do not necessarily reflect the T cell population in liver tissue. However, for a life-threatening disease like ACLF, barriers existed to get liver biopsy for such severe diseases. Nevertheless, the overall features of peripheral lymphocyte subsets in ACLF shown in this study are valuable and could help to narrow the ranges in further studies.

## Conclusion

The abnormal distribution of circulating lymphocytes probably associated with the progressive development of HBV-related ACLF. The decrease in CD8^+^ T cell counts may be related to poor prognosis in HBV-ACLF patients. Our findings will contribute to a further understanding of the immune pathogenesis of HBV-related ACLF.

## Data Availability Statement

The raw data supporting the conclusions of this article will be made available by the authors, without undue reservation.

## Ethics Statement

The studies involving human participants were reviewed and approved by Ethics Committee of the First Affiliated Hospital of Xi'an Jiaotong University. The patients/participants provided their written informed consent to participate in this study.

## Author Contributions

JL and Y-LH planned and designed the study and wrote the protocol. Y-LH, T-YC, and Y-RZ were responsible for the treatment of patients. C-HH, YC, M-MZ, Z-JG, and M-JF participated in the study monitoring and management. JL, J-ZL, and JW were biostatisticians and participated in the data analysis and writing of the report. All authors read and approved the final version of the work.

## Conflict of Interest

The authors declare that the research was conducted in the absence of any commercial or financial relationships that could be construed as a potential conflict of interest.

## Publisher's Note

All claims expressed in this article are solely those of the authors and do not necessarily represent those of their affiliated organizations, or those of the publisher, the editors and the reviewers. Any product that may be evaluated in this article, or claim that may be made by its manufacturer, is not guaranteed or endorsed by the publisher.
